# Age-Related Cataract, Cataract Surgery and Subsequent Mortality: A Systematic Review and Meta-Analysis

**DOI:** 10.1371/journal.pone.0112054

**Published:** 2014-11-04

**Authors:** E Song, Hongpeng Sun, Yong Xu, Yana Ma, Hong Zhu, Chen-Wei Pan

**Affiliations:** 1 Lixiang Eye Hospital of Soochow University, Suzhou, China; 2 School of Public Health, Medical College of Soochow University, Suzhou, China; Zhongshan Ophthalmic Center, China

## Abstract

**Purpose:**

Changes in lens may reflect the status of systemic health of human beings but the supporting evidences are not well summarized yet. We aimed to determine the relationship of age-related cataract, cataract surgery and long-term mortality by pooling the results of published population-based studies.

**Methods:**

We searched PubMed and Embase from their inception till March, 2014 for population-based studies reporting the associations of any subtypes of age-related cataract, cataract surgery with all-cause mortality. We pooled the effect estimates (hazards ratios [HRs]) under a random effects model.

**Results:**

Totally, we identified 10 unique population-based studies including 39,659 individuals at baseline reporting the associations of any subtypes of cataract with all-cause mortality from 6 countries. The presence of any cataract including cataract surgery was significantly associated with a higher risk of death (pooled HR: 1.43, 95% CI, 1.21, 2.02; *P*<0.001; I^2^ = 64.2%). In the meta-analysis of 9 study findings, adults with nuclear cataract were at higher risks of mortality (pooled HR: 1.55, 95% CI, 1.17, 2.05; *P* = 0.002; I^2^ = 89.2%). In the meta-analysis of 8 study findings, cortical cataract was associated with higher risks of mortality (pooled HR: 1.26, 95% CI, 1.12, 1.42; *P*<0.001, I^2^ = 29.7%). In the meta-analysis of 6 study findings, PSC cataract was associated with higher risks of mortality (pooled HR: 1.37, 95% CI, 1.04, 1.80; P = 0.03; I^2^ = 67.3%). The association between cataract surgery and mortality was marginally non-significant by pooling 8 study findings (pooled HR: 1.27, 95% CI, 0.97, 1.66; P = 0.08; I^2^ = 76.6%).

**Conclusions:**

All subtypes of age-related cataract were associated with an increased mortality with nuclear cataract having the strongest association among the 3 cataract subtypes. However, cataract surgery was not significantly related to mortality. These findings indicated that changes in lens may serve as markers for ageing and systemic health in general population.

## Introduction

Visual impairment (VI) is a global health burden, associated with increased mortality in different ethnic groups [1]–[5]. Age-related cataract, including nuclear, cortical and posterior subcapsular (PSC) cataract, is a leading cause of visual impairment worldwide [6]–[13]. Although age-related cataract seems to increase the risk of death, the relationship of different cataract subtypes and mortality remains unclear. It is important for clinicians and ophthalmologists to understand the associations between different cataract subtypes and mortality as the pathophysiology, treatment and impact on visual functioning of the 3 subtypes are different [14]. A clearer understanding of the relationship between cataract subtypes and mortality may provide further insights into the pathogenesis of these conditions. In addition, if a specific subtype of cataract is associated with increased mortality, lens imaging targeting this subtype may be a useful tool for the assessment of systemic health and ageing of the whole body. This research question is also of great importance from public health perspectives regarding the increasing burden of cataract throughout the world. The question of whether age-related cataracts are associated with an increased risk of death or whether the association between increased mortality and the occurrence of the cataract is caused simply by the relationship with higher age is unclear.

To address this gap, we conducted a systematic review and meta-analysis to examine the association between different subtypes of age-related cataract, cataract surgery and long term mortality from available population-based studies.

## Methods

### Ethnics Statement

The study was approved by the Ethics Committee, Medical College of Soochow University and followed the tenets of the Declaration of Helsinki.

### Search Strategy and Inclusion Criteria

The method for performing this meta-analysis is similar with our previously work on overweight/obesity and cataract [15]. We performed this systematic review and meta-analysis to examine the association of different subtypes of age-related cataract, cataract surgery and mortality based on the Meta-analysis of Observational Studies in Epidemiology guidelines [16]. We searched the electronic databases of PubMed and Embase for relevant papers reporting the longitudinal association of age-related cataract, cataract surgery with all-cause mortality published up to March, 2014 with the following search terms (formatted for PubMed search): ((“vision, low”[MeSH Terms] OR (“vision”[All Fields] AND “low”[All Fields]) OR “low vision”[All Fields] OR (“visual”[All Fields] AND “impairment”[All Fields]) OR “visual impairment”[All Fields] OR “vision disorders”[MeSH Terms] OR (“vision”[All Fields] AND “disorders”[All Fields]) OR “vision disorders”[All Fields] OR (“visual”[All Fields] AND “impairment”[All Fields])) OR (“cataract”[MeSH Terms] OR “cataract”[All Fields]) OR (“cataract”[MeSH Terms] OR “cataract”[All Fields] OR (“lens”[All Fields] AND “opacity”[All Fields]) OR “lens opacity”[All Fields]) OR (“cataract extraction”[MeSH Terms] OR (“cataract”[All Fields] AND “extraction”[All Fields]) OR “cataract extraction”[All Fields]) OR (“cataract extraction”[MeSH Terms] OR (“cataract”[All Fields] AND “extraction”[All Fields]) OR “cataract extraction”[All Fields] OR (“cataract”[All Fields] AND “surgery”[All Fields]) OR “cataract surgery”[All Fields])) AND ((“mortality”[Subheading] OR “mortality”[All Fields] OR “mortality”[MeSH Terms]) OR (“mortality”[Subheading] OR “mortality”[All Fields] OR “survival”[All Fields] OR “survival”[MeSH Terms]) OR (“death”[MeSH Terms] OR “death”[All Fields])). Titles and abstracts of the studies were independently reviewed by 2 authors (ES and HPS). All duplicate articles were removed. Inconsistencies during the literature search were resolved by consensus and the reference lists of all identified studies were screened.

We included studies if they were population-based, reported any subtypes of age-related cataract or cataract surgery as an independent variable and all-cause mortality as the outcome measure. The Age-Related Eye Disease Study [1] is a multi-center intervention trial rather than a population-based observational study, which has reported the association between major age-related eye diseases and mortality. As the study participants covered a wide geographical areas and the sample size was large, we feel the results should be included in the meta-analysis. We only included studies in which age-related cataract was assessed based on standardized protocols. Furthermore, we included studies only if the summary estimates such as the relative risk (RR) or hazards ratios (HR) with 95% confidence interval (CI) were reported in the paper, or allowed for calculation based on the data presented in the paper. We summarized the measures of association as HR for all the studies. We excluded studies if they were not population-based, did not have standardized cataract grading, or were published in languages other than English. If one study reported two results at different follow-up periods, the result with a longer follow-up period was included in the analysis.

### Data Extraction and Assessment of Study Quality

For each study in the analyses, information on first author, publication year, study name, study location, ethnic group, sample size at baseline, age range of the study participants at baseline, follow-up periods, definitions of age-related cataract and mortality, summary estimates and corresponding 95% CI, and confounding factors adjusted for were extracted and recorded in a form designed previously.

The study quality were assessed using the tool described by Sanderson et al [17]. The variables examined included the methods for selecting study participants, methods for measuring exposure (age-related cataract or cataract surgery) and outcome variable (all-cause mortality), design-specific sources of bias, methods for controlling confounding, statistical methods and conflict of interest.

### Statistical Methods for the Meta-analysis

The meta-analysis was performed using Stata version 12.0 (StataCorp, College Station, TX). We meta-analyzed the fully-adjusted study-specific risk estimates under random effects model, accounting for both within and between study variability. All-cause mortality was treated as the outcome measure while nuclear, cortical, PSC cataract or cataract surgery were analyzed as the independent variable. Statistical heterogeneity among studies was evaluated using I^2^ Statistic [18]. Values of 0 to 24%, 25% to 49%, 50% to 74%, and more than 75% denote no, low, moderate, and high heterogeneity, respectively [19]. Publication bias was assessed using the Egger regression asymmetry test and the Begg's test.

## Results

### Literature Search and Characteristics of Included Studies

**Figure 1:**
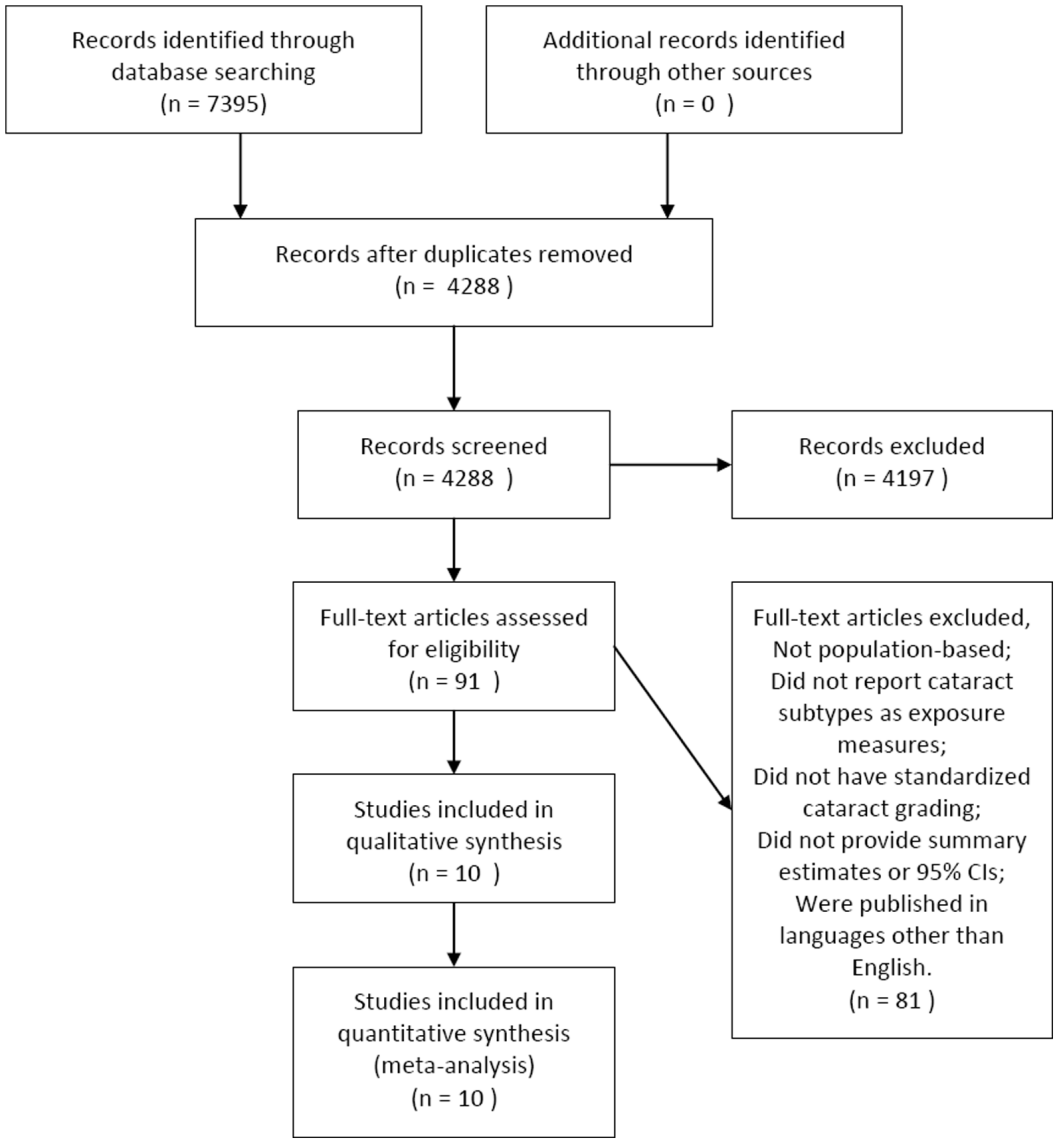
Flow diagram showing the selection process for inclusion of studies.

The literature search yielded 4288 unique titles from the two electronic databases. In total, 91 articles were retrieved for full-text review, 12 of which met all the predefined inclusion criteria. However, two studies reported 2 results at different follow-up periods [2], [5], [20]–[21] and only the result with a longer follow-up period were included. Finally, 10 unique population-based studies [1]–[2],[21]–[28] were included in this meta-analysis. (**Figure 1**)

Characteristics of the 10 studies included in the meta-analysis are summarized in **Table 1**. Among the 10 studies, 2 were conducted in Asia [22]–[23], 2 were in Australia [21], [24], 2 were in Europe [25]–[26] and the remaining 4 were in the United States [1]–[2], [27]–[28]. The 10 cohort studies comprised a total of 39,659 individuals at baseline. The sample sizes at baseline ranged from 860 to 6,339. The follow-up periods ranged from 2 to 14 years. The range of mortality rates in all identified studies was very wide (from 3.2% to 32%), which may be due to methodological issues including various ages of enrollment at baseline, follow-up periods and ethnic groups.

**Table 1:** Characteristics of the included studies.

Author (year)	Study Name	Location	Ethnicity	Sample Size at baseline	Age at baseline (years)	Follow up period (yrs)	Incidence of mortality: N (%)
Knudtson et al (2006)	The Beaver Dam Eye Study	United States	Whites	4926	43–84	14	1576 (32)
Nucci et al (2004)	The Priverno Eye Study	Italy	Italians	860	45–69	7	44 (5.1)
Clemons et al (2004)	The Age-Related Eye Disease Study	United States	Whites	4753	55–81	9	534 (11)
Cugati et al (2007)	The Blue Mountains Eye Study	Australia	Whites	3654	≥49	11	1051 (28.9)
Hennis et al (2000)	The Barbados Eye Study	United States	Blacks	4709	40–84	4	306 (6.8)
Borger et al (2003)	The Rotterdam Study	Netherlands	Whites	6339	≥55	7	1359 (21.4)
West et al (2004)	The Salisbury Eye Project	United States	Whites and Blacks	2520	65–84	2	147 (5.8
Xu et al (2009)	The Beijing Eye Study	China	Chinese	4439	≥40	5	143 (3.2)
McCarty et al (2001)	Melbourne Visual Impairment Project	Australia	White	3271	40–98	5	231 (7.1)
Khanna et al (2013)	The Andhra Pradesh Eye Diseases Study	India	Indians	4188	≥30	11	799 (19.1)

### Quality Assessment of the Included Studies

All studies graded cataract based on standardized protocols such as the Wisconsin grading system, the Wilmer grading system or the Lens Opacity Classification System [29]–[31]. In most of the included studies, mortality data were reviewed and retrieved from official records or hospital medical records. All studies adjusted for age in the multivariate analysis; however, diabetes, the most important confounding factor was not adjusted for in one study. All studies gave loss to follow-up rates and described sampling methods in the baseline examinations, albeit in varying degrees. However, only 6 studies outlined specific exclusion criteria and provided information on non-responders. Conflicts of interest were not reported in all included studies. The detailed assessment of study quality of the included study is described in **Table 2**.

**Table 2:** Assessment of methodological quality of included studies.

Study	Methods for selecting study participants	Methods for measuring exposure (cataract)	Methods for measuring outcome (mortality)	Design-specific sources of bias	Methods for controlling confounding, and statistical methods	Conflict of Interest
Knudtson et al (2006)	4926 persons aged from 43 to 84 years in the period from September 15, 1987, to May 4, 1988, participated in the baseline examination of the population-based Beaver Dam Eye Study and were followed by about 10 years.	Wisconsin Grading System	National Death Index was used for matching against national death data.	Lost to follow up bias; survival bias; chance finding; residual confounding	Adjustments for age, sex, proteinuria, history of cancer, BMI, ratio of total to high-density lipoprotein cholesterol level, smoking, pulse rate,diabetes status, cardiovascular disease history, sedentary lifestyle, education, and systolic blood pressure were made using Cox proportional hazards models	None reported
Nucci et al (2004)	860 legal residents of Priverno represented 70% of a random sample selected to participate in the Di.S.Co. Project—a population-based study on cardiovascular risk factors carried out by the Italian National Institute of Health.	Slit-lamp examination	Review of municipality records	Lack of statistical power; lost to follow up bias; survival bias; chance finding; residual confounding	Adjusting for age, gender, diabetes, serum cholesterol, high-density lipoprotein cholesterol, and cardiovascular diseases using Cox proportional-hazards regression model.	None reported
Clemons et al (2004)	A total of 4757 persons aged 55 to 81 years at enrollment were entered into the study at 11 clinical centers between 1992 and 1998 and were followed up by 9 years	The Age-Related Eye Disease Study (AREDS) system	Hospital records and death certificates.	Lost to follow up bias; chance finding; residual confounding	Cox proportional-hazards regression model adjusting for age, sex, race, education, smoking status, body mass index, diabetes mellitus, angina, cancer, and hypertension	None reported
Cugati et al (2007)	Australian adults aged 49 years and older at baseline in the Blue Mountains area were followed up for about 11 years	Wisconsin Grading System	The Australian National Death Index data	Lost to follow up bias; survival bias; chance finding; residual confounding	Cox regression models assessed associations between cataract and mortality risk during 11 years after adjusting for age, sex, BMI, hypertension, diabetes mellitus, current smoking, and history of stroke, angina, or myocardial infarction	None reported
Hennis et al (2000)	The studies were based on a simple random sample of Barbados African Americans, 40 to 84 years old. 4631 persons completed baseline examinations at the study site. Surviving members of the cohort were invited to return for a 4-year follow-up visit.	Lens Opacities Classification System II	Ministry of Health records	Lost to follow up bias; survival bias; chance finding; residual confounding	Cox proportional-hazards regression. age; gender; self-reported history of diabetes, cardiac disease, and stroke, or combination of these major illnesses; family history of diabetes and hypertension; body mass index; waist-to-hip ratio; alcohol use; and cigarette smoking.	None reported
Borger et al (2003)	A prospective cohort study of all residents aged 55 years and older from a suburb of Rotterdam	Lens Opacities Classification System II	Municipal registry and medical records.	Lost to follow up bias; chance finding; residual confounding	Cox proportional hazard regression analysis, adjusted for appropriate confounders (age, gender, smoking status, body mass index, cholesterol level, atherosclerosis, hypertension, history of cardiovascular disease, and diabetes mellitus)	None reported
West et al (2004)	A random sample of 2520 residents of Salisbury aged 65 to 84 years was recruited for a home interview and an examination at the SEE clinic. Two-year follow-up was conducted.	Wilmer grading scheme.	Hospital records and the National Death Index	Lost to follow up bias; survival bias; chance finding; residual confounding	Logistic regression model adjusting for age, sex, smoking, diabetes, cormorbid conditions and body mass index.	None reported
Xu et al (2009)	At baseline in 2001, the Beijing Eye Study examined 4439 subjects with an age of 40 years or more. In 2006, all study participants were invited for a follow-up examination.	The Age-Related Eye Disease Study (AREDS) system	Death certificate, hospital medical records	Lost to follow up bias; survival bias; chance finding; residual confounding	Logistic regression was used to investigate the associations of the binary dependent variable mortality with the continuous or categorical independent variables.	None reported
McCarty et al(2001)	Cluster random sampling was employed to identify nine pairs of census collector districts in the Melbourne statistical division from which to recruit eligible residents. Baseline examinations were conducted between 1992 and 1994. In 1997, 5 year follow up examinations of the original cohort commenced.	Wilmer grading scheme.	National Death Index	Lack of statistical power, lost to follow up bias; survival bias; chance finding; residual confounding	Multivariate logistic regression adjusting for age, sex, country of birth, smoking, hypertension, arthritis, best corrected visual acuity, age related maculopathy, glaucoma, uncorrected refractive error,diabetes,gout and cardiovascular disease.	None reported
Khanna et al (2013)	A large-scale prevalence survey of blindness and visual impairment was conducted between 1996–2000 on 10,293 individuals of all ages in three rural and one urban clusters in Andhra Pradesh, Southern India. More than a decade later, participants in rural clusters were traced to determine ocular risk factors for mortality.	Wilmer grading scheme.	Questionnaire	Lack of statistical power, lost to follow up bias; survival bias; chance finding; residual confounding	Cox-proportional hazard model after adjusting for age, gender, diabetes, hypertension, body mass index, smoking and education status	None reported

### Age-related cataract and mortality

**Figure 2:**
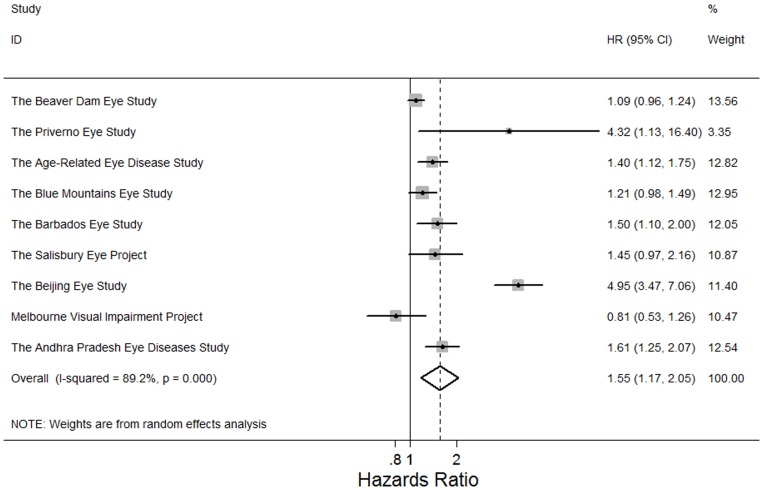
Random effects meta-analysis investigating the association between nuclear cataract and all-cause mortality. HR = hazards ratio; CI = confidence interval.

**Figure 3:**
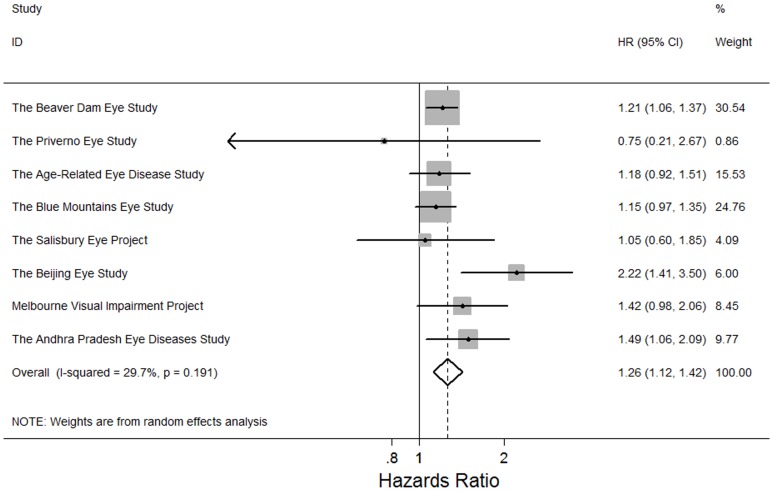
Random effects meta-analysis investigating the association between cortical cataract and all-cause mortality. HR = hazards ratio; CI = confidence interval.

**Figure 4:**
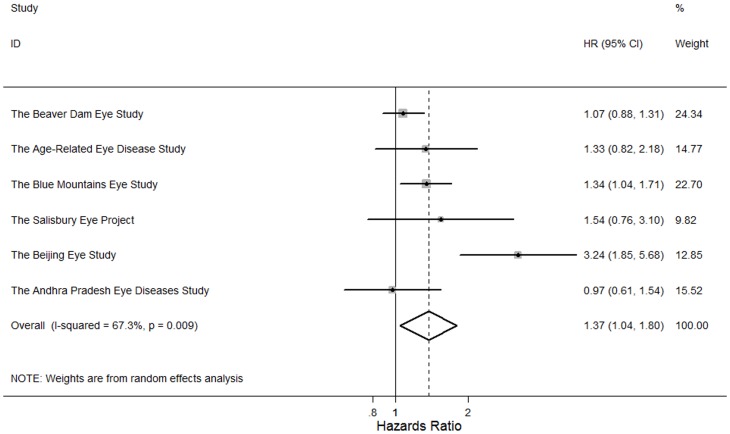
Random effects meta-analysis investigating the association between posterior subcapsular cataract and all-cause mortality. HR = hazards ratio; CI = confidence interval.

**Figure 5:**
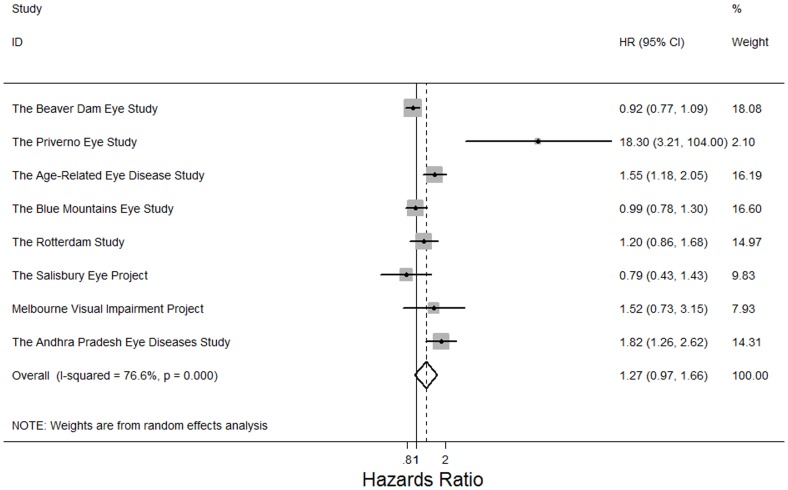
Random effects meta-analysis investigating the association between cataract surgery and all-cause mortality. HR = hazards ratio; CI = confidence interval.

Adults with any cataract or cataract surgery previously were more like to die (pooled HR: 1.43, 95% CI, 1.21, 2.02; *P*<0.001; I^2^ = 64.2%). In the meta-analysis of 9 study findings, adults with nuclear cataract were at higher risks of mortality (pooled HR: 1.55, 95% CI, 1.17, 2.05; *P* = 0.002; I^2^ = 89.2%) (**Figure 2**). In the meta-analysis of 8 study findings, cortical cataract was associated with higher risks of mortality (pooled HR: 1.26, 95% CI, 1.12, 1.42; *P*<0.001, I^2^ = 29.7%) (**Figure 3**). In the meta-analysis of 6 study findings, PSC cataract was associated with higher risks of mortality (pooled HR: 1.37, 95% CI, 1.04, 1.80; P = 0.03; I^2^ = 67.3%) (**Figure 4**). The association between cataract surgery and mortality was marginally non-significant by pooling 8 study findings (pooled HR: 1.27, 95% CI, 0.97, 1.66; P = 0.08; I^2^ = 76.6%)(**Figure 5**). After excluding the Salisbury Eye Project with a follow-up period of only 2 years, all cataract subtypes were still associated with mortality (data not shown).

**Table 3** shows the association between cataract and mortality by follow-up years of the identified studies and cataract diagnosis. The estimates were more robust in studies with longer follow-up periods or more standardized grading systems (graded by lens photographs).

**Table 3:** Association between cataract and mortality by follow-up years and cataract diagnosis.

	Nuclear cataract				Cortical Cataract				Posterior Subcapsular Cataract				Cataract Surgery			
	N	HR	95% CI	I2 (%)	N	HR	95% CI	I2 (%)	N	HR	95% CI	I2 (%)	N	HR	95% CI	I2 (%)
**Follow-up years**																
More than 5 years	5	1.32	1.09, 1.60	68.0	5	1.2	1.10, 1.32	0	4	1.16	1.01, 1.33	0	6	1.33	0.98,1.80	82.1
No more than 5 years	4	1.73	0.84, 3.56	93.6	3	1.52	1.03,2.25	55	2	2.31	1.12, 4.77	61.9	2	1.06	0.56,2.00	45.6
**Cataract diagnosis**																
By photographs	7	1.50	1.09, 2.06	91.5	7	1.27	1.13, 1.44	36	6	1.37	1.04, 1.80	67.3	6	1.2	0.92, 1.56	74.8
By Ophthalmologist clinically	2	2.07	0.80, 5.35	56.3	1	0.75	0.21, 2.67	-	0	-	-	-	2	4.08	0.29, 58.07	89

N = number of studies RR = hazards ratio CI = confidence interval.

There was no evidence of publication bias as indicated by a non-significant Egger test (all *P*>0.05) and Begg's test (all *P*>0.05) in all analyses.

## Discussion

In this systematic review and meta-analysis of population-based studies, all subtypes of age-related cataract were associated with increased mortality with nuclear cataract having the strongest association among the 3 cataract subtypes. However, cataract surgery was not significantly related to mortality. These findings indicated that changes in lens may serve as markers for ageing and systemic health in the general population.

Our meta-analysis confirmed the longitudinal association between nuclear cataract (nuclear sclerosis of the lens) and increased mortality reported in previous studies. In addition, we found that the association of nuclear cataract with increased mortality was strongest among all of the three cataract subtypes. Nuclear sclerosis of the lens may be a marker for changes in structural proteins of the whole body, mainly resulting from oxidation that cause diminished function [32]. For example, the levels of glutathione (GSH), one of the most important antioxidant agents of the lens, are found to be much higher in the outer cortex fiber cells where GSH is synthesized [33]. Reduced GSH reaches the lens nucleus by diffusion from the surface fiber cells through an abundant network of gap junctions [34]. With increasing age, a greater percentage of GSH is oxidized in the nucleus, making it more susceptible to oxidation [35]. Chronic oxidative stress, especially in brain and immune cells, could accelerate the rate of ageing, leading to an increased mortality in adults with nuclear cataract.

Compared with nuclear cataract, we found a weaker but significant association between cortical cataract and less survival. In the Blue Mountains Eye Study, the association between cortical cataract and increased mortality was no longer significant in persons without diabetes, indicating that the observed association may be confounded by the presence of diabetes [5]. Diabetes is one of the most consistent determinants for cortical cataract [36]–[38]. In this systematic review, most of the included studies have adjusted for the effect of diabetes in the multivariate analysis (**Table 2**), indicating that the association between cortical cataract and mortality may be, at least partially, independent. UV light exposure could increase the risk of cortical cataract [39]. Exposure to UV light, a known risk factor for cortical cataract, can lead to oxidative stress in the lens, which is a main cause for cortical cataract. This process is similar with other systemic causes of oxidative stress, which may increase the risk of systemic morbidity [40].

The association of PSC cataract and mortality was also positively significant in this meta-analysis although there were fewer studies reporting this association compared with other subtypes. At current stage, we were unable to provide a reasonable biological explanation for this observed association. Animal studies have demonstrated that injection of peroxidative substances into the vitreous resulted in the formation of PSC cataract [41], which may be mediated by peroxidative damage of the whole body.

We found a marginally non-significant association between cataract surgery and mortality, which may be a balanced effect of aged-related cataract and good knowledge of health. Adults who have undergone cataract surgery certainly had cataract previously, which may increase the risk of long term mortality. On the other hand, they tend to have better knowledge of health and healthier lifestyle compared with those who did not. For example, in the Beaver Dam Eye Study, adults with cataract surgery were more likely to take vitamin supplements [2]. Also, adults with previous cataract surgery may have better access to health care services. A clinical trial investigating the effect of cataract surgery on survival in cataract patients may be useful to confirm this finding.

Our meta-analysis may have some important clinical implications. Previous studies have shown that age-related cataract is associated with systemic comorbidities [42] and general functional decline [43] in older adults. We confirmed that these systemic comorbidities and functional decline are sufficient to cause death in the general population. Therefore, we propose that lens imaging may be a necessary clinical examination for normal heath screening of older adults and should not be limited only in eye clinics. Currently, there are various technologies [44]–[45] for lens imaging and it remains controversial which one is the best to quantify lens opacity in elderly adults.

There are several strengths of the meta-analysis. The qualities of the included studies were relatively high with population-based samples achieving reasonable follow-up rates and the use of standardized grading systems for age-related cataract. Limitations of this meta-analysis should also be acknowledged. First, the potential biases in the original studies, methodological issues and different strategies for adjusting for confounders could affect the results from this meta-analysis. The longitudinal association of age-related cataract and mortality may have been confounded by other unadjusted factors or selection bias. Second, different studies used different cataract grading system, sampling strategies, follow-up period, and ways of mortality measures, which would affect the estimates of mortality. These differences could have also contributed to the observed high heterogeneity across studies. Thirdly, most included studies only reported the association between cataract and all-cause mortality and therefore we were unable to summarize the association between cataract and the cause of mortality. Fourthly, the number of the contributing prospective studies was small and therefore, detailed subgroups analysis may not be feasible. Finally, although there was no evidence of publication bias as indicated by a non-significant Egger test and Begg's test, publication bias could still be of concern because studies that report statistically significant results are more likely to get published than studies that report non-significant results, and this could have distorted the findings of our meta-analyses.

In conclusion, this systematic review and meta-analysis confirmed a longitudinal associations of all age-related cataract subtypes but not cataract surgery with increased mortality. The association was strongest for nuclear cataract. Clinically, ophthalmologists and clinicians should be aware that lens imaging may be a useful tool for the assessment of ageing of the whole body.

## Supporting Information

Checklist S1 **PRISMA checklist.**
(DOC)
